# Parental knowledge, attitudes, and practices towards childhood fever among South-East and East Asian parents: A literature review

**DOI:** 10.1371/journal.pone.0290172

**Published:** 2023-09-08

**Authors:** Hoi Lam Ng, Huiyuan Li, Xiaohuan Jin, Cho Lee Wong

**Affiliations:** 1 Faculty of Medicine, The Nethersole School of Nursing, The Chinese University of Hong Kong, Hong Kong, China; 2 The School of Nursing, Nanjing University of Chinese Medicine, Nanjing, China; Universiti Kebangsaan Malaysia, MALAYSIA

## Abstract

**Aim:**

The aim of this literature review was to identify, summarize, and critically appraise available empirical articles on the knowledge, attitudes, and practices towards childhood fever management among South-East and East Asian parents.

**Design:**

A literature review following PRISMA.

**Methods:**

Articles were limited to those available in the English language. Articles had to be empirical studies that used a qualitative or quantitative research design with full-text available; focus on parental knowledge, attitudes, and practices towards fever; and be published in South-East and East Asia. Searches were conducted with CINAHL, PubMed and Scopus from inception to June 2022, and eleven articles were included after removing duplicates and excluding irrelevant articles.

**Results:**

Narrative synthesis was conducted according to four themes: source of fever information, knowledge level, attitudes, and practices towards childhood fever. Parents showed different fever knowledge needs and various information-seeking behaviors. A low level of fever knowledge was revealed in terms of temperature, fever causes, potential harms and influencing factors. South-East and East Asian parents mainly reported anxiety, concerns and fever phobia. Fever assessment methods and fever management strategies varied based on parents’ cultural background and beliefs.

**Conclusions:**

The findings of this review highlight that inadequacy of fever knowledge and negative attitudes towards childhood fever exist in South-East and East Asian parents. Parents have diverse cultural practices during their children’s febrile episodes. However, some of them conflict with current medical guidelines, as they prioritize fever and body temperature reduction. This raises questions about their effectiveness and safety. Although some of them are medically discouraged, there are others that have been proven beneficial for the symptomatic relief of childhood fever. The results indicate an urgent need to develop a cultural-sensitive educational intervention for childhood fever management among South-East and East Asian parents. Unified educational interventions are needed to address parental concerns and fever-related knowledge needs.

## Background

Fever is a temporary elevation of body temperature above the average daily range of 36.6°C–38°C, as measured by a rectal thermometer [1,2]. It is a common symptom experienced by almost every child at some point. Fever can be an indicator of benign (e.g., the common cold) or severe conditions (e.g., lethal diseases and meningitis) and is usually self-limiting in children [2]. However, even in mild cases, most parents seek information about fever management and worry about the potentially severe consequences of fever, such as seizures, brain damage, and even death, although these outcomes are rare [2], leading to heightened anxiety.

Despite being common, a recent systematic review of 36 studies on information needs related to childhood fever found that parents have a low level of knowledge about fever [3]. Furthermore, a review of scientific literature indicated that parental knowledge regarding the definition and management of fever is deficient [4]. Parents rarely define fever correctly and tend to have misconceptions regarding fever and engage in practices which differ from recommendations [5–7].

Parental knowledge greatly influences attitudes and fever management in their children [8]. A lack of knowledge regarding the pathophysiology and management of fever is an essential driver of fever phobia among parents; this can cause parents to become overly concerned about the height of the fever, how quickly the fever rises, the appearance and behavior of their child, and the underlying cause of the fever [9]. Parents’ inadequate knowledge about fever may also lead to unnecessary and inappropriate treatments, such as being unaware of the correct frequency of administering antipyretics at incorrect doses or intervals, which can increase healthcare-seeking behavior [10,11]. These practices can negatively affect children’s health, such as toxicity from supratherapeutic doses [12].

Parents’ unscientific and irrational attitudes towards fever can significantly impact the management of childhood fever [13,14]. Although appropriate levels of anxiety in parents are paramount to promoting health-protective behaviors in febrile children, which include close monitoring of symptoms and increasing fluid intake, studies have found that many parents (57%–68%) exhibit moderate to high anxiety levels during their children’s febrile episodes [15–18]. These findings align with the long-lasting phenomenon of fever phobia, which refers to an “unrealistic fear of fever expressed by parents” [19]. Parents’ fever-related anxiety and concern often lead them to practice non-evidence-based strategies to reduce temperature, which can cause further stress for children and parents [14] and increase emergency room visits [20]. A study in Hong Kong showed that caregivers of paediatric patients with fever symptoms were more than twice as likely to consult more than one doctor during an illness episode without a referral [21]. These actions inflict adverse psychological and financial consequences for families and burden the healthcare system unnecessarily [21].

Strategies for childhood fever management can vary in different countries. Thompson et al. conducted a first systematic review on childhood fever, and the results showed that treatments ranged from supportive care at home to seeking assessment in the emergency department [3]. Using antipyretic medications, including acetaminophen and ibuprofen alone, in combination or alternating, was the most common response to a febrile episode, with adjunct fever management practices, such as light clothing and sponge baths, also being adopted. Other literature views indicated that parents’ conceptualizations of fever in children and information-seeking behaviors in fever management differ according to country of origin [8,22]. However, most studies and reviews have focused on Western or global populations [3,8,22], ignoring the impacts of cultural factors on parents’ management of fever in different countries, especially cultural beliefs in South-East and East Asian countries. Growing evidence suggests that racial differences and cultural beliefs can influence parents’ attitudes and management approaches to fever in children [3,18,23]. One of the examples is alternative medicine, which is greatly influenced by traditional values. It has a different perspective on the pathophysiology and treatments of febrile diseases compared to modern Western medicine. For instance, in Traditional Chinese Medicine, fever is associated with warm-pathogen diseases involving the Taiyin meridian, and consuming Chinese herbal medication and a light diet are recommended to restore body equilibrium [24]. On the contrary, conventional medicine focuses on managing the accompanying symptoms rather than targeting the fever itself and temperature reduction [10].

Unfortunately, the use of alternative medicine for fever management remains understudied. In a systematic review of 74 national and international guidelines regarding childhood fever management, only five studies investigated the use of alternative medicine, while most focused on conventional treatments such as antipyretic drugs and water baths [10]. However, some traditional practices based on cultural beliefs (i.e., South-East and East Asia) may contradict the principle of modern Western medicine and harm children. For instance, some parents in Singapore give spirit water to their febrile children, but the effect is unknown [15]. This highlights the need for further research on the potential benefits and drawbacks of alternative approaches to fever management.

To date, no scientific synthesis of the literature has been conducted, and the evidence regarding the knowledge, attitudes, and practices of South-East and East Asian parents towards childhood fever remains unclear. Data from Western populations may not represent the Asian region due to cultural differences. Therefore, it is essential to summarize the literature regarding fever management by South-East and East Asian parents regarding their knowledge, attitudes, and practice and improving scientific fever management for children outside the hospital setting.

This literature review aimed to identify and summarize the evidence related to knowledge, attitudes, and practices of South-East and East Asian parents towards fever in healthy children. The results may guide the development of future research and education programs specific to this population Asian parents.

## Methods

This review followed the Preferred Reporting Items for Systematic Reviews and Meta-Analyses (PRISMA) guidelines (S1 File).

### Objectives

This literature review aimed to identify, summarize, and critically appraise current evidence on (1) South-East and East Asian parents’ knowledge, attitudes, and practices towards fever in healthy children and (2) factors associated with parents’ knowledge, attitudes, and practices towards fever.

### Literature search

Three English language databases (CINAHL, PubMed, and Scopus) were searched from inception to June 2022. The search terms include ‘parents’, ‘children’, ‘fever’, ‘knowledge’, ‘attitude’, and ‘practice’ were combined in each database using free-text terms and Medical Subject Headings (MeSH) where available. A sample search strategy in CINAHL is illustrated in the S2 File. A manual search of the references of the included articles was conducted to locate additional relevant articles.

### Inclusion criteria

Articles were included if they (1) were empirical studies that used a qualitative or quantitative research design with full-text available; (2) focused on parental knowledge, attitudes and practices towards fever and (3) were published in East Asia (China including Hong Kong, Macau, and Taiwan, Mongolia, Japan and South Korea) or South-East Asia (Brunei, Myanmar, Cambodia, Indonesia, Laos, Malaysia, the Philippines, Singapore, Thailand, Timor-Leste and Vietnam).

### Exclusion criteria

The following items were excluded: (1) studies focusing on other febrile symptoms, e.g. febrile convulsion and febrile seizure; (2) editorials, letters, case reports or commentaries; (3) in vivo or in vitro studies without human data and (4) conference abstracts or poster abstracts without full-text publication. Articles conducted in countries from the Middle East, South Asia, and Central Asia were also excluded because of economic, cultural, and social differences. Diverse religious beliefs can also significantly influence social norms, values, and traditions, bringing differences in medical practices.

### Study selection

Two investigators (NHL and WCL) reviewed the search results independently on three successive levels. (1) the article titles were initially screened to find the potential studies relevant to this review’s objectives (title stage). (2) The abstracts of these articles were then further reviewed (abstract stage). (3) In the final stage, the full texts of the remaining articles were reviewed based on the inclusion and exclusion criteria (full-text stage). Any discrepancies were discussed with the third reviewer (LH) to reach a consensus.

### Quality appraisal

The primary investigator (NHL) evaluated the selected articles and cross-checked them by a coinvestigator (LH) using the Joanna Briggs Institute Critical Appraisal Tool (JBI) for cross-sectional studies [25], which was used to criticise cross-section study designs. The JBI critical appraisal checklist for cross-sectional studies includes eight items to assess inclusion criteria, study sample, measurements, confounding factors, and statistical analysis. Each item was evaluated using four responses: yes, no, unclear or not applicable. Disagreements were solved by consulting a third reviewer (CLW) to reach a consensus. A global rating of each study was examined by combining all component ratings (Table 1). No studies were excluded based on the quality assessment ratings.

**Table 1:** Summary of studies with critical appraisal using joanna briggs institute checklist.

Appraisal questions	Ayuningtyas et al. (2020) [32]	Bong & Tan (2018) [33]	Chang et al. (2013) [27]	Chang et al. (2012) [36]	Dong et al. (2015) [28]	Hew et al. (2018) [34]	Kwak et al. (2013) [35]	Sakai et al. (2009) [29]	Sakai & Marui (2009) [29]	Sakai et al. (2012) [30]	Soon et al. (2003) [15]
1. Were the criteria for inclusion in the sample clearly defined?	Yes	Yes	Yes	Yes	Yes	Yes	Yes	Unclear	Unclear	Unclear	Yes
2. Were the study subjects and the setting described in detail?	Yes	Yes	Unclear	Yes	Yes	Unclear	Yes	Yes	Yes	Yes	Yes
3. Was the exposure measured in a valid and reliable way?	Not/Applicable	Not/Applicable	Not/Applicable	Not/Applicable	Not/Applicable	Not/Applicable	Not/Applicable	Not/Applicable	Not/Applicable	Not/Applicable	Not/Applicable
4. Were objective, standard criteria used for measurement of the condition?	Yes	Yes	Yes	Yes	Yes	Yes	Yes	Yes	Yes	Yes	Yes
5. Were confounding factors identified?	Not/Applicable	Not/Applicable	Not/Applicable	Not/Applicable	No	Not/Applicable	Not/Applicable	No	No	Not/Applicable	Not/Applicable
6. Were strategies to deal with confounding factors stated?	Not/Applicable	Not/Applicable	Not/Applicable	Not/Applicable	No	Not/Applicable	Not/Applicable	Yes	Yes	Not/Applicable	Not/Applicable
7. Were the outcomes measured in a valid and reliable way?	Unclear	Yes	Yes	Yes	Unclear	Yes	Unclear	Unclear	Unclear	Unclear	Unclear
8. Was appropriate statistical analysis used?	Yes	Yes	Yes	Yes	Yes	Yes	Yes	Yes	Yes	Yes	Unclear
Overall appraisal	Include	Include	Include	Include	Include	Include	Include	Include	Include	Include	Include

### Data extraction

Data were extracted by one investigator (NHL) and checked for accuracy by the other investigator (LH) independently. Discrepancies were resolved through discussion. The following details were summarized from all articles: author(s), year, region of research, study design, study setting, data collection method, eligibility/recruitment, sample size, characteristics of caregivers, characteristics of children and synthesis themes.

### Data synthesis

Data synthesis is the process of integrating findings from the included articles. A narrative synthesis was used in this review to examine the study findings from the articles. A narrative synthesis framework was adopted, including the following steps: (1) developing a preliminary synthesis of findings of included articles, (2) exploring relationships in the data, and (3) assessing the robustness of the synthesis [26].

## Results

**Fig 1:**
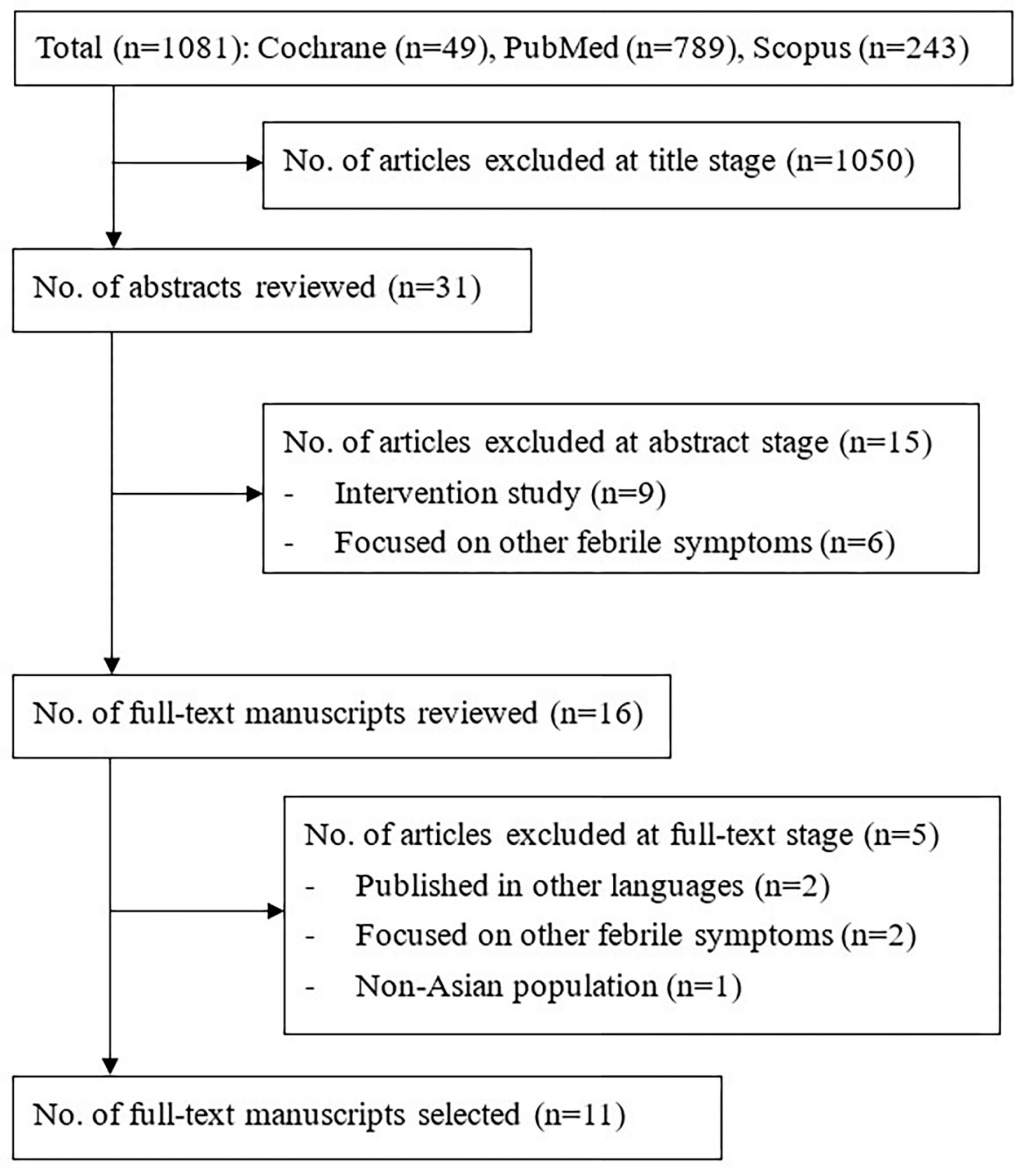
Literature searching history.

After applying the inclusion criteria and excluding the duplicated articles, 11 articles were selected from the three English electronic databases. An article selection flow chart is presented in Fig 1. These studies were conducted in different South-East and East Asian countries including China (n = 3), Japan (n = 3), Malaysia (n = 2), Indonesia (n = 1), Korea (n = 1) and Singapore (n = 1) (Table 2). All of the included studies were quantitative designs involving 3,429 participants. Ten included studies adopted convenience sampling, while one adopted purposive sampling. All studies collected cross-sectional data with either a self-administered survey or a structured interview questionnaire. Critical appraisal information is detailed in Table 1.

**Table 2:** Included studies characteristics.

Authors	Year	Region of Research	Study Design	Study Setting	Data collection method	Eligibility/ Recruitment	Sample Size	Characteristics of caregivers	Characteristics of children	Synthesis themes ^a^ 1 2 3 4
Ayuningtyas et al.	2020	Indonesia	Quantitative Cross-sectional	1 public place	Closed questionnaire consisted of 16 questions about maternal knowledge about fever in preschool children with no information about reliability and validity.	Mothers who have preschool children aged 3–6 years. Mother’s job as a medical paramedic personnel/ who has beliefs regarding the management of fever or illness in children were excluded	N = 109	Age range: 45.9% 29–34 y.o. 30.3% 35–40 y.o. 23.9% 23–28 y.o. 78% high school/ equivalent education level 12.8% middle school level 2.8% elementary school level 6.4% higher education/ college level 77.1% housewives 15.6% general employees 2.8% civil servants 4.6% not working	Children aged 3–6 years	2 4
Bong & Tan	2018	East Malaysia	Quantitative Cross-sectional	1 maternal and child health clinic	English and Malay version of the knowledge and concerns regarding childhood fever adapted from a Taiwanese version (satisfactory internal consistency reliability with a Cronbach’s alpha of 0.7).	Parents of children who visited the maternal and child health clinic and were literate in Malay or English	N = 157	81.5% mothers 17.8% fathers 0.7% not stated - Parents were not involved in caring excluded - Mean age: 30.4 years	Aged 6 months to 6 years Children with serious chronic medical diseases excluded	2 3 4
Chang et al.	2013	Taiwan, China	Quantitative Cross-sectional	61 Paediatric clinics, 9 kindergartens	Self-developed research questionnaire in Chinese to assess knowledge of fever, parental concerns, fever management, and sources of information about fever. (The content validity index was 0.95; face validity: 90% participants indicated the questionnaire was easy to understand; reliability: Item discrimination analysis)	Parents of children who visited the paediatric clinics. Parents of children in one class from each kindergartens which are randomly selected.	N = 649	82.8% mothers 17.2% fathers - Mean age: 33.15 years	Aged less than 6 years	1 2 3 4
Chang et al.	2012	Taiwan, China	Quantitative Cross-sectional	1 medical centre	Cross-sectional survey using a self-designed questionnaire in three aspects: demographic data; the fever management approaches prior to admission; the knowledge and the comprehension about administration of antipyrexial medication (acetaminophen syrup) (The content validity index was 0.91. The Cronbach’s alpha was 0.94, and the Kuder–Richardson 20 was 0.87)	Primary caregivers of children with: (1) symptoms of fever (rectal temperature up to 38oC and above); (2) newly admitted (within 24 hours); (3) six years old and under	N = 102	Mean age: 35.8 years 79.2% mothers 14.7% grandmothers 5.9% other 16.7% < = 25 years 43.3% 25.1 to 35 years 23.3% 35.1 to 45 years 16.7% >45 years 43.2% less than a high-school education 56.8% complete high school or above 54.9% Mandarin-speaking Chinese 35% Aboriginal 4.9% immigrant (3 Vietnamese and 2 Indonesians)	22.5% < = 1 years 48% 1.1–3 years 29.5% 3.1–6 years Diagnosis: 73.5% respiratory infection 26.5% others	1 4
Dong et al.	2015	China	Quantitative Cross-sectional	1 paediatric outpatient department	A Chinese version of re-administrated questionnaire after content validation, including caregivers’ knowledge about fever, causes of fever, possible effects of fever, concerns, management, and source of information.	Caregivers of children who visited the paediatric outpatient department and the wards	N = 621	51% from inpatient ward 49% from outpatient department 79.4% mothers 16.8% fathers 3.8% grandparents - Caregivers with experience working in healthcare institutions and hospitalisation were excluded	Aged less than 3 months were excluded	1 2 3 4
Hew et al.	2018	Malaysia	Quantitative Cross-sectional	12 different public places	Self-developed questionnaire based on previous studies. Face and content validity were completed. The Cronbach’s alpha of the questionnaire was > 0.7.	Local Malaysian parents aged ≥ 18 years with at least one child aged ≤ 6 years, who can communicate in English	N = 430	64.9% mothers 47.4% Malay 39.8% Chinese 10.7% Indian - Mean age: 35 years - Age above/equal to 18 years - Able to communicate in English	Aged less than/equal 6 years	2 3 4
Kwak et al.	2013	Korea	Quantitative Cross-sectional	6 tertiary referral hospitals	Self-developed questionnaire based on previous studies. The questionnaire included socioeconomic and demographic information, the children’s clinical history, knowledge of fever and antipyretics, and practice and management of febrile children. No information about validation.	Korean caregivers who brought a child to a participating emergency department due to febrile illness	N = 746	Mean age: 34.7 years 62.6% female 34.3% male 63.8% fathers 31.4% mothers 3.5% grandparents 1.3% other relatives 26.3% high school 70.6& college 3.1% unknown 10.7% less than 2000 US dollars 39.4% between 2000 to 3500 USD 27.5% between 3500 to 5000 USD 16.9% more than 5000 USD 5.5% unknown	Mean age: 2.8 years 33.4% only child 319% attended day-care centers 10.6% experienced febrile convulsion(s) 3.2% experienced hypothermia due to antipyretics	2 4
Sakai et al.	2009	Japan	Quantitative Cross-sectional	5 health urban health department	Anonymous, self-administered questionnaire in Japanese with no information about reliability and validity.	Parents of children who visited one of five health departments for a routine 18-month well-baby check during the time of the study. Only mothers’ response were analysed.	N = 386	- All mothers, other caregivers excluded to avoid confounders 11.4% febrile seizure group 88.6% non-febrile seizure group	Children with a routine 18-month well-baby check-up, with or without a history of febrile seizure	1 2 3 4
Sakai & Marui	2009	Japan	Quantitative Cross-sectional	5 health urban health department	Anonymous, self-administered questionnaire in Japanese with no information about reliability and validity.	Parents of children who visited one of five health departments for a routine 18-month well-baby check during the time of the study. Only mothers’ response were analysed.	N = 418	- All mothers, other caregivers excluded to avoid confounders 89.5% from nuclear families 51.7% with single child 48.3% with pleural children	Children with a routine 18-month well-baby check-up	1 2 4
Sakai et al.	2012	Japan	Quantitative Cross-sectional	1 University-based, paediatric, walk-in clinic	Anonymous, self-administered questionnaire in Japanese with no information about reliability and validity.	Parents of all children who visited the paediatric clinic at the time of the study. Exclusion of children in critical condition.	N = 211	85.3% mothers - Mean age: 36.1 years	5.7% aged 0–3 months 46.9% aged 3–36 months 47.4% older than 36 months 19.4% health maintenance visit 29% acute visits without fever 23.7% acute visits with fever 19% for follow-up visits	1 2 3 4
Soon et al.	2003	Singapore	Quantitative Cross-sectional	1 polyclinic	Interview-based questionnaire in English, Mandarin and Malay with no information about reliability and validity.	Parents of children aged between 3 months and 5 years, who were brought to the polyclinic for a well- or sick- visit	N = 557	78.1% mothers 16.5% fathers 5.4% both parents 70.4% Chinese 16.7% Malay 10.2% Indian 2.7% other ethnic groups	81.1% between 3 and 36 months 18.9% older than 36 months	1 2 3 4

^a^ Synthesis themes:1 Source of fever knowledge; 2 Knowledge level; 3 Attitudes towards childhood fever; 4 Practices towards childhood fever.

### Narrative synthesis

The following four themes were established after applying the synthesis framework: (1) source of fever knowledge, (2) knowledge level, (3) attitudes towards childhood fever and (4) practices towards childhood fever (Tables 2 and 3).

**Table 3:** Summary of the source of knowledge, knowledge, attitudes, and practices in diverse South-East and East Asian populations.

Authors	Region/Country	Source of Knowledge	Knowledge	Attitudes	South-East and East Asian Parents’ fever Practices
Ayuningtyas et al., 2020 [32]	Indonesia		**Knowledge level**: 21.1% good 16.5% enough 62.4% less		**Percentage of different levels of fever management practices**: 2.8% good 16.5% enough 23.9% less
Bong & Tan, 2018 [33]	East Malaysia		**Knowledge score**: Normally distributed with a mean score of 10.03 ± 3.6 39.5% knew the correct temperature of fever 80.9% knew that fever is an immune reaction 71.3% had the misconception that fever causes diseases 86% believed that fever is due to an imbalance of heat and cold in the body 93.6% believed that fever could cause harm to children	72% reported always being worried Main reasons for parental concern: 68.8% the discomfort of the child 68.2% persistently rising body temperature 63.7% feared harms of fever The feared harms of fever that worried the parents: 67.5% seizure 52.2% brain damage 44.6% mental incapacity 38.9% death **Participants’ concerns** were mainly influenced by: 59.9% own previous experience 42.0% family member’s previous experience 39.5% not knowing the cause 35.7% doctor’s advice upon consultation	26.1% had good knowledge of fever management 56.7% believed that it is necessary to treat fever regardless of body temperature 92.4% would administer fever medication to treat feared fever complications
Chang et al., 2013 [27]	Taiwan, China	Source of information: 89.1% medical literature 53.8% popular books, newspapers, and magazines 50.7% relatives and friends Desired source of information: 80% medical literature 50.4% popular books, newspapers, and magazines 41.9% public-health-sponsored courses 37.3% media Perceived important information: 81.5% fever management 74% causes of fever 50% benefits and risks as a biological mechanism	Median participant score: 10/23 (IOR = 8, 12) 47.2% did not completely understand the information provided by healthcare providers 41.6% dissatisfied with the information provided **Possible complications of fever**: 77.7% brain damage 65.6% seizure 55.5% lowered intelligence 8.4% pneumonia 17.6% death 14.8% disability	**Level of concern**: 57.1% always worried 29.5% frequently worried 12.5% sometimes worried	83.7% want immediate antipyretic treatment 50% took their child to a medical clinic within 24 hours of fever onset 89.5% gave antipyretics before the clinic visit **Refer to Western medical management**: 77.6% ice packs 60.2% providing sports drinks 52.2% providing warm drinks **Traditional Chinese medical management**: 18.2% “cold” drinks 8.2% “hot” drinks 6.2% Shou Jing 3.0% Gua Sha 2.2% Chinese herbal medicine
Chang et al., 2011 [36]	Taiwan, China	Source of information: 72.5% health care professionals 31.4% internet			**Most preferred method for reducing fever**: 65.7% using antipyretic syrup 60.8% giving antipyretic via rectal suppository 95.1% had previously administered acetaminophen syrup 46.4% responded with the correct medication dose 66.0% knew the duration of taking medication, answering ‘four–six hours’ 65% did not know the maximum dosage during 24-hour period 90.2% of the caregivers were unaware of the possibility of liver toxicity
Dong et al., 2015 [28]	China	Source of information: 48.3% doctors and nurses 28.8% parent’s books 25% experience 24.6% TV or newspaper 22.6% internet 20.3% neighbors or relatives	**Threshold of fever**: 9.8% considered 37°C 39.8% considered 37.5°C 59.2% considered 38°C. **High fever threshold**: 56.4% considered as 39°C 27.2% considered 38.5°C 7.6% considered 40°C **Potential effects predicted**: 74.7% brain damage 38.6% death 21.9% convulsion 14% deafness 11.9% blindness	**Level of worrisome**: 1.1% not worried at all 20.1% a little worried 44.4% very worried 34.3% extremely worried	**Frequency of temperature checking**: 20.6% > 2 h 32.7% every 1 to 2 h 33.3% every 30 min to 1 h 11.8% every 15 to 30 min. 1.0% < 15 min **Antipyretics used**: 79.4% ibuprofen 9.5% acetaminophen 4.3% the method of combining or alternating ibuprofen and acetaminophen 6.8% other choices, mostly Chinese traditional medicine **The way of getting antipyretics**: 33.8% drugstore 61.4% hospital 4.8% both drugstore and hospital **Method of physical cooling**: 46.2% Tepid sponging 19.5% alcohol sponging 15% cold toweling 10.5% fever cooling patch 2.7% cold sponging
Hew et al., 2018 [34]	Malaysia		**Perception about fever**: 34% identified 38 °C as the threshold of fever 45% as a symptom of certain illnesses 36% as a process of natural child growth 19% as a disease itself	A high parental burden was observed as evidence by the parental fever management scale scores, which ranged from 16 to 35 (mean 27.84 ± 4.11)	86% used thermometers to measure their child’s body temperature 81.4% used antipyretics to manage fever if there were no comorbid symptoms 70% seek a doctor’s help if there were any comorbid symptoms **Source of medicine**: 80.9% directly from a doctor 7.0% from a pharmacist 8.8% used leftover medicine 3.0% used leftover medicine from child’s siblings 67% sleep in the same room as the child 67% take a febrile child to a doctor 55.6% check on them at night 49.8% taking temperature (49.8%), 53.3% wanting to know their child’s temperature 40.2% waking the febrile child for an antipyretic at night
Kwak et al., 2013 [35]	Korea		**Temperature of clinically significant fever**: 69.2% equal to or higher than 38°C **Threshold of febrile temperature** 13.8% less than 38.3 14.2% less than 37.5°C 48.3% believed the body temperature could reach higher than 42.0°Cwithout treatment 10.5% believed the body temperature could reach higher than 44°C **Possible adverse effects**: 39.5% brain damage 8.8% unconsciousness 7.8% loss of hearing/vision		Worries about improbable adverse events: 41.9% resistance to antipyretics 39.7% over-dependency **Body temperature measurement interval**: 40.0% 30 min 24.0% 1–2 hr 22.1% 15 min **Fever control**: 93.0% tepid bath/massage at home 81.8% used lukewarm water, 6.2% cold water 1.6% hot water 92.8% checked the body temperature of the child before giving an antipyretic agent 66.2% guardians woke the child to give antipyretics. 47.5% followed a 4-hr scheduled interval for administering the medication 29.4% intermittently practiced such alternating use of antipyretic agents If the child remained febrile after the appropriate dose of antipyretic agent: 49.3% give a tepid bath/massage 35.7% visited the hospital
Sakai et al., 2009 [29]	Japan	Source of information: Doctor (FS group: 90.9%; non-FS groups: 90.6%)	The mean body temperature of fever threshold was 37.8°C in both FS and non-FS groups. **Possible complications of fever**: Febrile seizure (FS group: 77.3%; non-FS group, 57.3%) Brain damage (FS group: 27.3%; non-FS group: 44.4%) **The mean body temperature causing complications**: 38.9°C in the FS group 39.2°C in the non-FS group		**Temperatures That Determine Antipyretic Use**: FS group: 27.3%; non-FS group: 33.3% do not rely on the height of fever to determine antipyretic use FS group: 25.0%; non-FS group: 15.5% do not use antipyretics **Reasons for Antipyretic Use**: To relieve the discomfort of fever (FS, 52.3%; non-FS groups, 58.8%) To prevent seizures (FS group, 34.1%; non-FS group, 18.7%) To prevent the disease from worsening (FS group, 11.4%; non-FS group, 26.6%) FS group: 27.3%; non-FS group: 33.3% do not seek treatment based on the height of their child’s fever
Sakai & Marui, 2009 [29]	Japan	Sources of information: 90.4% doctor 46.9% reading 41.4% own parents or parents of spouse	**Temperatures considered to indicate fever**: mean = 37.8°C 97.4% believed that fever results in some complication **Possible complications of fever**: 69.4% dehydration 58.9% febrile seizure 43.3% brain damage **Temperatures that cause complications**: mean temperature = 39.2°C		**Management of fever at home**: 88.0% give fluids 54.1% cool the head 23.2% cool the body 13.6% use antipyretics 11.7% keep the body warm **Temperatures that determine whether to consult a healthcare provider**: 33.9% did not based on the height of their child’s fever **Temperatures that determine antipyretic use**: 32.3% do not rely on the temperature 23.0% do not use antipyretics **Reasons for antipyretic use**: 58.3% To relieve the discomfort of fever
Sakai et al., 2012 [30]	Japan	Source of information: 92% doctors and nurses 28% experience 21% friends and relatives 16% books 13% internet	**Definition of fever**: 62% <37.8°C 38% 37.9–38.9°C How high a fever can go if left untreated: 33% 40.7–43.2°C **Possible complications of fever** 28% seizure 20% brain damage 4% dehydration	**Level of worrisome**: 47% very worried 53% somewhat worried	**Frequency of temperature checking**: 38% >120 min 29% 61–120 min 26% 31–60 min Temperature above which parents would give antipyretics: 2% below 37.8% 44% below 38.8%
Soon et al., 2003 [15]	Singapore	The most important perceived source of knowledge: medical staff (doctors and nurses)	48.6% considered a temperature equal to or less than 37.8°C to be a high fever 4.3% believed the temperature could rise to 43.3°C or above if left untreated. **Perceived harmful consequences**: 68.8% brain damage 3.2% death	50.4% were very worried 91.5% were worried that fever could possibly cause harmful effects **Possible harmful effects**: 68.8% brain damage 14.1% seizure 3.4% death 49.9% worried more if their child had a high temperature than if their child looked sick	70.5% would check their child’s temperature every hour or less 24.8% would administer paracetamol for temperatures equal or less than 37.8°C 85.8% would give antipyretics before the temperature reached 38.9°C 7.4% consult the traditional medical practitioner 11.1% administer traditional medicine concomitantly with the polyclinic consultation **Fever management practices**: 80.6% sponging 55.1% showering the child 27% switching off the air conditioner 11.8% switching off the fan 5.8% wrapping or bundling their child Traditional practices: 7.4% would consult traditional doctors as well as Western doctors for their child’s fever 11.1% would give traditional herbal remedies to their children 4.5% give talisman water for drinking 1.4% put oil on the child’s fontanelle 1.3% put special herbs on the child’s hands and feet

### Source of fever knowledge

Six studies investigated parental information-seeking behavior toward childhood fever [15,27–31]. Healthcare professionals, namely doctors and nurses, were found to be parents’ most common source of information in five studies [15,27–31]. Although medical literature was reported as the primary source in Chinese (Taiwan) research, most of the participants (80%) indicated that they would like to receive medical information from healthcare professionals [27]. Other information sources included spouses, friends and relatives, courses, media, books, own parents, parents from spouses and the internet [27–31].

The desired fever information included management practices (81.5%), causes (74%), symptoms (65%) and benefits and risks of fever as a biological mechanism (50%) [27].

### Knowledge level

Ten studies assessed parental knowledge levels, and all of them reported low levels of parental knowledge regarding childhood fever [15,27–35]. Studies in East Malaysia and Indonesia found that only 26.1% and 21.1% of parents had good knowledge about childhood fever, respectively [32,33]. Similarly, the median knowledge score was only 10 of 23 in Chinese (Taiwan) research [27]. Although the majority of parents (74%–80.9%) could correctly identify the cause of fever (as an immune response), some of them attributed it to exposure to wind and cold (32.4%, [27]) and an imbalance of heat and cold within the body (15.6%–86%) [27,33]. Nearly two-thirds of Malaysian parents (71.3%) believed that fever causes diseases [33].

#### Temperature

Regarding the definition of fever, a significant proportion of parents (48.6%–100%) could not correctly identify a febrile temperature. Except for the Korean study that reported the lowest incorrect rate at 14.2% [35], Japanese parents had the highest false rate, which ranged from 62% to 100%, as reported in two studies [29,30]. In addition, a small proportion of Singaporean (4.3%), Korean (10.5%) and the majority of East Malaysian (84.7%) parents believed that fever could rise to 43.3°C or infinitely if left untreated [15,33,35].

#### Cause of fever

Only two studies investigated parental knowledge regarding the cause of fever [27,33]. Although most parents correctly identified it as an immune response, some still had different misconceptions. For instance, parents in Malaysia and Taiwan would explain fever from the perspective of Chinese medicine, e.g., the imbalance of heat and cold [27,33].

#### Potential harm

Almost all parents (91.4%–98%) in the studies believed that childhood fever could bring about harm, including brain damage, febrile seizure, dehydration, loss of hearing and vision or even mortality [15,27–31,33,35]. Of note, brain damage (20%–77.7%) and febrile seizure (28%–67.5%) were mainly reported [15,27–31,33,35]. Only a tiny proportion of Japanese parents (2.6%–2.8%) believed that fever would not harm children [29,30]. Most parents thought these complications needed to be triggered by elevated temperatures in three Japanese studies (threshold <40°C) [29–31].

#### Influential factors

Factors that influenced parental knowledge levels were also investigated. Family demographics, such as age, education level, job and monthly income, were associated with parental knowledge of fever [27,29,31,32,34]. For instance, higher education levels and careers as professionals and management were associated with higher knowledge levels in Taiwan, China [27]. Furthermore, ethnicity and religion also affect parents’ knowledge, as Indians and Chinese were reported to better understand childhood fever than other ethnicities [33,34]. Parents with no religion had a higher knowledge level than those who believed in Buddhism [27]. Results also found that mothers had a better understanding than fathers [27].

### Attitudes towards childhood fever

#### Types of attitudes

Six studies investigated parental attitudes during a child’s febrile episode and found that most of the parents expressed a high level of anxiety and concern [15,27,28,30,33,34]. The majority of Asian parents reported moderate to high levels of anxiety (47%–86.6%) [15,27,28,30,33]. Of note, in In China, Japan, and Singapore, nearly half of the parents reported being “very worried”, while over one-third of the Chinese parents (34.3%) even reported being “extremely worried” [15,28,30]. In addition, a significant proportion of Malaysian (72%) and Chinese (Taiwan) parents (86.6%) were found to have the highest concern level [27,33]. The results illustrated that the phenomenon of fever phobia also exists in South-East and East Asia.

#### Influential factors

Bong and Tan [33] explored factors influencing parents’ anxiety levels. High anxiety was associated with low parental knowledge levels [27]. It also reported that discomfort of children (68.8%), persistently rising body temperature (68.2%) and fear of harm (63.7%) were the chief reasons for parental concern [33]. Their concerns were mainly influenced by their own or a family member’s previous experience with child fever (59.9% and 42%, respectively), not knowing the cause of the fever (39.5%) and doctor advice upon consultation (35.7%) [33].

### Practices toward childhood fever

#### Assessments

Five studies investigated fever assessment methods performed by parents [28,29,34–36]. Most Malaysian parents (86%) reported using a thermometer to assess their children’s temperature, while others reported using a touching technique [34]. Regarding sites of measurement, assessment strategies varied in different countries. In Malaysia and Korea, the eardrum thermometer was the most commonly used instrument, while the auxiliary was the preferred site of the Japanese [30,34,35]. Both methods were popular in China [36]. Nearly all parents in China (99.4%) and Japan (100%) would check their children’s temperature at regular intervals [28,30]. The majority of Singaporean (70.5%) and Korean parents (62.1%) would check their child’s temperature every hour or less [15,35].

#### Management strategies

A wide variety of fever management practices, which aimed to relieve discomfort, promote sleep, and prevent brain damage and seizure, were used by Japanese parents [30,31]. Common practices included encouraging fluid intake, tepid sponging, and cooling the head and body [15,27–31,36].

In addition, traditional medicine and folk treatments were applied. For instance, some Taiwanese parents in China adopted traditional Chinese medicine techniques and Taiwanese folk remedies, e.g., ingestion of “cold” and “hot” drinks, “Shou Jing”, “scraping, Gua sha”, and intake of Chinese herbal medicine [27]. However, some traditional interventions were adopted by parents [37]. For example, some Japanese parents warm their children’s bodies, while a small proportion of Chinese parents would apply cold and wine sponging [28,29]. The combined therapy of traditional treatment and modern Western treatment were also found. Some Singaporean parents consult a conventional medical practitioner, and 11.1% administer traditional medicine concomitantly with polyclinic consultation [15].

Practices regarding the use of antipyretics varied in different regions. Most parents would administer antipyretics to their children in febrile conditions [15,28–31,33,34,36]. Reasons for antipyretic use included relieving the discomfort, promoting sleep, and preventing brain damage, seizures, and deterioration of diseases [29,31]. Only a minority of Taiwanese parents (14.2%) reported that they would not give antipyretics to their children because they did not want to disrupt doctors’ evaluations [27]. Common concerns included drug resistance (41.9%) and over-dependency (39.7%) [35].

Seven studies analysed the use of antipyretics concerning the degree of temperature [15,28–31,35,36]. Nearly all parents in Korea would check their child’s body temperature before giving an antipyretic medication [35]. Nevertheless, about one-third (33.9%) of parents in Japan did not rely on it to decide on the use of antipyretics [31]. Most parents responded that a temperature >38°C was the timing of giving medications [15,29,31,36]. Only a minority (2%–40%) of parents reported giving antipyretics at 38°C [15,28,30,36].

Nearly three-quarters of the Korean parents recognised the trade names of the antipyretics used, while only a few of them (9.4%) knew their generic names [35]. The most common antipyretics chosen by Chinese parents were ibuprofen (79.4%), followed by acetaminophen (9.5%) [28]. If the child remained febrile after having the antipyretic agent, common management adopted by parents included a tepid bath, visiting the hospital, alternating to diclofenac sodium suppository and readministering the same medication [35,36].

Chang et al. [36] further explored the common misunderstandings of parents toward antipyretics, which included side effects of overdose possibility (e.g., liver toxicity), the maximum number of doses during a day, medication dose and duration of taking medication. Grandmothers, immigrant mothers, and parents with lower academic qualifications and older age were found to have higher misconception rates [36]. Over one-third of the parents misunderstood the drug package insert instructions and medication envelope instructions, which were attributed to the tiny words printed and the incomprehension of Chinese [36]. It highlighted the insufficient knowledge of using antipyretics among parents.

#### Influential factors

Several factors affected parental management practices regarding childhood fever. For instance, Korean parents with an only child tended to seek medical attention more than those with more than one offspring [35]. Parents worried about brain damage would check the child’s body temperature more frequently [35]. Of note, parents with poor knowledge of fever showed a four times chance of showing poor management strategies compared to those with good knowledge [32].

## Discussion

This study is the first review summarising parental knowledge, attitude, and management regarding childhood fever in South-East and East Asia. The results highlight the unmet childhood fever information needs, inadequacy of fever knowledge, negative attitudes, and diverse fever management practices in South-East and East Asian parents. Our results indicated that educating them about childhood fever and providing psychological support are warranted for better decision-making on childhood fever management at home.

### Parents’ knowledge about fever

Consistent with previous Western studies [3], this review suggests that South-East and East Asian parents generally have insufficient knowledge about childhood fever, including diverse perspectives on febrile temperature and inadequate understanding of potential harm. Although different questionnaires were used across studies, making head-to-head comparisons was difficult because of the various definitions of fever concerning the site of thermometer measurement [38]. Nevertheless, the current study’s results consistently show that most parents could not identify febrile temperature correctly. The correct rate of fever temperature identification often ranged from ~0% to 50%, comparable to another systemic review (19%–45%) [3]. The results highlight the lack of basic understanding of childhood fever for most South-East and East Asian parents. The possible reasons could be related to the information provided by healthcare professionals, traditional perspectives on the cause of fever, and the influential demographic factors.

On the one hand, the current review demonstrates that healthcare professionals are parents’ most common source of information. It offers an excellent opportunity for them to deliver appropriate and effective education during clinical consultation while preventing parents from being exposed to unreliable information sources. However, some misconceptions (e.g., higher temperatures create more significant risks to the child) might be unintentionally reinforced through health consultations, especially when healthcare professionals ask about body temperatures. As such, temperature-focused questioning coupled with parents’ misconceptions or fears may create a false sense of importance associated with numerical fever values rather than the child’s overall well-being [3]. Therefore, it is valuable to standardize the knowledge about childhood fever for healthcare professionals in the paediatric department, incorporating the health guidelines on fever management in their own country, which will help ensure the accuracy of parents’ understanding of childhood fever.

On the other hand, certain traditional beliefs (i.e., the perspective of Chinese medicine on explaining fever) [27,33] may mislead parents into focusing on the level of fever instead of its origin (i.e., infection); it may lead to excessive temperature monitoring and overusing antipyretics [15]. Therefore, additional studies are warranted to promote parental knowledge about fever’s origin in different South-East and East Asian countries, providing insights for further education programs. Thus, the parents’ abilities to identify fever should be enhanced [33].

In addition, studies reported that immigrants, old age, and low education levels were associated with a higher level of the misconception of fever and its management [27,29,31,32,34,36]. Therefore, health literacy is suggested to be taken into consideration. Hospitals and paediatric clinics, and primary healthcare clinics can be an ideal platform for healthcare professionals to deliver tailored education concerning parents’ abilities and needs. Developing accessible and easy-understanding educational interventions about childhood fever will benefit parents of different socio-demographic characteristics and levels, realizing their information-seeking. Hence, the impact of low health literacy can be minimised.

### Parents’ attitude towards fever

The current review demonstrates that the anxiety levels of South-East and East Asian parents towards childhood fever are comparable to Western studies. The majority of Asian parents reported moderate to high levels of anxiety (47%–86.6%), which is consistent with findings from another systematic review (57%–68%) [3,15,27,28,30,33]. It reveals that fever phobia also exists in the South-East and East Asian population. Of note, a significant proportion of parents in Malaysia (72%) and China (Taiwan, 86.6%) expressed the highest level of concern [27,33]. The reason behind the disparities in anxiety levels in different countries can be further investigated. Nevertheless, among all the perceived harms of childhood fever, South-East and East Asian parents were concerned more about brain damage and mental retardation [15,16,27,28,33,35,37,39,40]. These findings are more apparent in China, Singapore and Malaysia. The possible reason is that South-East and East Asian parents, especially Chinese, emphasise children’s academic attainment, which heavily relies on their intelligence level [15]. Therefore, they would worry more about their children’s intellectual development. Moreover, due to cultural impacts (e.g., endurance/control in Chinese Confucian culture), some parents tend to take excessive control of fever to alleviate their anxiety, such as alternatively using antipyretic drugs, ignoring children’s health, and thus aggravating their anxiety [8]. This phenomenon could be further explored, hence contributing to a theoretical framework for a culturally sensitive educational scheme to address parents’ concerns in this area. Studies to compare groups from different social and cultural backgrounds are also paramount to identify the factors influencing fever phobia.

Of note, a study revealed that doctors’ advice upon consultation contributed to parents’ concerns, indicating that information provided may also heighten their anxiety level [33]. In this regard, in addition to giving education, healthcare professionals should also focus on the psychological status of parents. Reassuring parents and reducing their mental burdens are essential. Otherwise, elevated anxiety levels may lead to the chain reactions of over-treatment and over-consultation, which are undesirable outcomes for the family and healthcare system. Psychological support designed in the future is suggested to emphasize the correct understanding of childhood fever and its harmfulness, focusing on the child’s well-being rather than using irrational ways to relieve their concerns and increase children’s burdens.

### Parents’ practices toward fever

Being influenced by cultural-bounded thoughts, parents in different countries share distinct cultural responses to fever. Some of them would adopt alternative medicine and seek advice from traditional medical practitioners instead of relying only on Western medicine. For instance, Taiwanese parents in China applied conventional Chinese medicine and Taiwanese folk remedies at home, such as the ingestion of “cold” drinks and “hot” drinks, “Shou Jing”, and “Gua Sha". This could be deeply influenced by the tenets of traditional Chinese medicine [27]. Singaporean parents would give talisman water for their children to drink and put oil on their fontanelles. This may be influenced by the inconsistent advice from information resources under the multicultural population in Singapore [15]. In contrast, Western medicine upholds a different viewpoint regarding fever management practices. Treatments focus on reduction of distress rather than temperature [10]. For instance, guidelines suggested giving antipyretics only in cases of discomfort [41].

Despite the prevalence of traditional fever management practices, there is a lack of evidence regarding their effectiveness. For instance, the practice of giving talisman water to febrile children in Singapore has not been studied, and evidence related to the effectiveness of warming children’s bodies in Japan is also limited to a theoretical level [10,15,29]. Although it is believed to reduce the energy needed to develop fever and, thus, alleviate the discomfort of the febrile child, this theory has not been supported by any empirical studies [10]. Similarly, the effectiveness of Gua sha, a traditional Chinese practice, for fever discomfort is unclear as most studies focus on its use for sports injuries and pain relief [42]. Additionally, the potential harm associated, such as bruising and soreness, raises doubts about its worthiness for fever management [42]. Moreover, some traditional practices are discouraged by current medical guidelines, e.g., cold sponging and wine sponging by Chinese parents [10,28]. These practices can result in a mismatch between the hypothalamic set point and skin temperature due to manual cooling, leading to discomfort in the child due to peripheral vasoconstriction, metabolic heat production, and increased shivering [10]. The findings have highlighted the potential risks and consequences of various cultural practices.

On the contrary, some traditional management can indeed provide beneficial clinical effects. For instance, ingesting “cold’ and “hot” drinks can foster fluid consumption, alleviating discomfort during a febrile episode [10]. Some active ingredients in traditional Chinese medications, such as Bupleuri Radix (Chaihu) and Scutellariae Radix (Huangqin), have also been proven effective as antipyretic agents [43]. However, being aware of the drug-drug interactions between traditional remedies and western medications is crucial. Despite this, by recognizing the positive impacts of these widespread practices, healthcare providers can enhance treatment effects and improve parents’ adherence to the education given. Further research can focus on the effectiveness and contraindications of various cultural practices to facilitate their utilization.

As mentioned, healthcare professionals serve as the primary information source for parents. They inflict a significant impact on parents’ management practices. Chang et al. [27] found that Taiwanese parents in China were prone to use an ice pack as a management practice instead of promoting comfort, a misconception rooted in earlier acceptance by medical professionals. However, this practice still existed over the previous decade [27,37]. In addition, early Japanese nursing practice suggested that warming a febrile child could help manage the symptom [29,44]. Although it would raise the child’s body temperature, some Japanese parents still adhered to this practice [29]. This discrepancy could be related to the core goal of improving fever guided by healthcare professionals and the personal experience of parents. Compared to Chinese parents, Japanese parents tend to rely on their personal experiences to accurately control seizures by warming the body when their child develops a high fever [29]. In light of this, formulating a uniform, updated, evidence-based guideline for healthcare professionals in different countries is paramount to preventing inappropriate suggestions given to parents. It is also essential to implement tailored management strategies according to children’s physical condition, unmet parental needs and cultural issues.

### Limitations

The current literature review has some limitations. First, only studies published in English were included, and studies published in other languages were excluded. Nevertheless, the current review included studies from different countries, including China, Malaysia and Japan, which ensure the coverage of findings in various South-East and East Asian populations. Second, six studies showed low quality, resulting in a methodological weakness, especially on unreliable measurement. However, this study aimed to investigate the phenomenon of parental knowledge, attitude and management, and using a weaker quantitative design (cross-sectional study) may only bring a trivial limitation to the adequacy of the study results. Third, no qualitative studies were included in this review. The current review suggests an absence of qualitative studies conducted in the South-East and East Asian region. Therefore, further qualitative research is paramount to know about parental unmet information needs on childhood fever and the potential cultural issues influencing their beliefs, hence providing insights for developing a culturally sensitive educational frame.

### Research and practice implications

#### Implications for future research

The results of this review provide several meaningful implications for future research. Firstly, qualitative studies are warranted to explore the South-East and East Asian parent’s experience managing childhood fever and their perspective in their cultural contexts. Secondly, as the current study is only a literature review, it is suggested to systematically summarize the scientific measurements of fever and effective management strategies for childhood fever in South-East and East Asian countries. Thirdly, as the review indicated, parents’ access to information about childhood fever is multifaceted, especially through the internet and the media, so considering the convenience of the internet, designing a friendly-using online information platform about childhood fever will promote parents obtaining evidence-based information in real-time.

#### Implications for clinical practice

This review also indicates implications for clinical practice. As healthcare professionals are the primary source of information, and most parents would seek advice for managing childhood fever, they serve as an ideal platform for education with high credibility. Therefore, the results of the current study highlight the need to develop an educational framework to unify information, providing consistent knowledge and management approaches to parents. First, the theoretical knowledge about childhood fever must be regularly updated for clinical healthcare professionals in the paediatric department. Health education program services are highly recommended to be integrated into broader primary care systems. Second, culturally specific and evidence-based education programmes should equip parents with correct information, appropriate attitudes, and skills to manage mild to moderate fevers without emergency and medical consultation.

## Conclusion

The current literature review provides a comprehensive understanding of parental knowledge, attitude, and management of childhood fever among South-East and East Asian parents. The results found that a low state of parental knowledge and negative attitudes toward fever exists in South-East and East Asian parents. Nevertheless, differences in perception and management of childhood fever still exist in South-East and East Asia compared to other countries. Evidence-based approaches based on cultural contexts to effectively manage childhood fever in South-East and East Asian countries warrant further systematic investigation. Evidence-based information also deserves attention from healthcare professionals.

## Supporting information

S1 File PRISMA checklist. (DOCX)

S2 File A sample search strategy in CINAHL. (DOCX)
