# Supplementary material for: Parental knowledge, attitudes, and practices towards childhood fever among South-East and East Asian parents: A literature review
Source: PLoS One. 2023 Sep 8;18(9):e0290172. doi: 10.1371/journal.pone.0290172 (PMC10490995; doi:10.1371/journal.pone.0290172)
Supplement: S2 File — (DOCX) [file pone.0290172.s002.docx]

**MeSH terms used in Database: CINAHL Complete**

- S1 (MH "Body Temperature+")
- S2 (MH "Fever")
- S3 (MH "Fever of Unknown Origin")
- S4 febri*
- S5 fever*
- **S6 S1 OR S2 OR S3 OR S4 OR S5**
- S7 (MH "Caregivers")
- S8 (MH "Family")
- S9 (MH "Parenting")
- S10 (MH "Parents+")
- S11 ("care giver*" or caregiver* or carer* or guardian*)
- S12 (families* or family* or father* or mother* or parent*)
- **S13 S7 OR S8 OR S9 OR S10 OR S11 OR S12**
- S14 (MH "Attitude to Health")
- S15 (MH “ Parental Attitudes”)
- S16 (MH “Parental Behavior”)
- S17 (MH "Health Knowledge")
- S18 (MH "Information Seeking Behavior")
- S19 attitude*
- S20 belie*
- S21 concern*
- S22 experienc*
- S23 Impression
- S24 knowledg*
- S25 misconce*
- S26 opinion*
- S27 perce*
- S28 perspective*
- S29 prefer*
- S30 underst*
- S31 view*
- **S32 S14 OR S15 OR S16 OR S17 OR S18 OR S19 OR S20 OR S21 OR S22 OR S23 OR S24 OR S25 OR S26 OR S27 OR S28 OR S29 OR S30 OR S31**
- S33 (MH "Adolescence+")
- S34 (MH "Child+")
- S35 (MH "Minors (Legal)")
- S36 (MH "Pediatrics+")
- S37 (adolescen* or boy* or child* or girl* or preschool* or school age* or schoolchild* or teen* or toddler*)
- S38 (baby* or babies or infant* or infancy or neonat* or newborn* or postmatur* or prematur* or preterm*)
- S39 p#ediatric*
- **S40 S33 OR S34 OR S35 OR S36 OR S37 OR S38 OR S39**
- **S41 S6 AND S13 AND S32 AND S40**
- S42 PT (commentary or editorial or letter)
- S43 S41 NOT S42
- S44 (TI "hay fever" OR AB "hay fever")
- S45 S43 NOT S44

**Combination of keywords:**

(“carers” or “caregivers” or “caretakers” or “guardians” or “parents’ or ‘parental”) AND (“children” or “childhood” or “paediatric” or “paediatric ”) AND (“febrile” or “fever” or “pyrexia”) AND (“attitude” or “belief” or “experience” or “impression” or “knowledge” or “perception” or “management” or “practice ”)
